# Supplementary material for: Induced Pluripotent Stem Cell-Derived Cardiomyocytes with SCN5A R1623Q Mutation Associated with Severe Long QT Syndrome in Fetuses and Neonates Recapitulates Pathophysiological Phenotypes
Source: Biology (Basel). 2021 Oct 18;10(10):1062. doi: 10.3390/biology10101062 (PMC8533193; doi:10.3390/biology10101062)
Supplement: Supplementary file 1 [file biology-10-01062-s001.zip › biology-1379995-supplementary.pdf]

Induced Pluripotent Stem Cell-Derived Cardiomyocytes with *SCN5A* R1623Q Mutation Associated with Severe Long QT Syndrome in Fetuses and Neonates Recapitulates Pathophysiological Phenotypes

Supplementary materials

1. Materials and methods

1.1. Supplemental Table S1: Primers used in sequencing and qPCR

Human Gene		Primer sequence (5′–3′)
SCN5A R1623Q sequence	F	TGCACAGTGATGCTGGCTGGAA
	R	CCAACAGCATGCTGTGCCTCTTC
h***GAPDH*** (qPCR)	F	AAGGTGAAGGTCGGAGTCAAC
	R	GGGGTCATTGATGGCAACAATA
h***TNNT2*** (qPCR)	F	TTCACCAAAGATCTGCTCCTCGCT
	R	TTATTACTGGTGTGGAGTGGGTGTGG
h***OCT4*** (qPCR)	F	GACAGGGGGAGGGGAGGAGCTAGG
	R	CTTCCCTCCAACCAGTTGCCCCAAAC
Mutagenesis	F	TCCCCGACGCTCTTCCAAGTCATCCGCCTGGCC
	R	GGCCAGGCGGATGACTTGGAAGAGCGTCGGGGA
Cloning of ***SCN5A*** exon 6a region	F	CAAGCATATGCTCGAGCACGACCCTCCACCCTGGACC
	R	CAGCCGGATCCTCGATCAGTCCCTCAGCTCCGAGTAGTT

1.2. qRT-PCR for absolute quantification

Human cardiac total RNA was purchased from Biochain Institute Inc. (CA, USA) and reverse-transcribed to complementary DNA (cDNA) using PrimeScript RT master mix (Takara Biotechnology Co., Ltd., Shiga, Japan). The expression of the target genes was measured using qRT-PCR with Universal SYBR Select Master Mix (Thermo Fisher Scientific, MA, USA) and a Pico Real PCR System (Thermo Fisher Scientific). The PCR conditions were as follows: initial activation at 50 °C for 120 s, initial denaturation at 95 °C for 120 s, followed by continued denaturation with 40 cycles of 95 °C for 15 s, and annealing at 60 °C for 60 s. The primers used in the qRT-PCR analysis are listed in Table S2. The housekeeping gene glyceraldehyde 3-phosphate dehydrogenase (*GAPDH*) was used as an internal control. The plasmids harboring the corresponding WT or neonatal *SCN5A* along with *GAPDH* cDNA fragments were linearized by appropriate restriction enzyme digestion, purified, quantified, and serially diluted in 1x TE

to produce a standard curve. Real-time PCR of standards and internal controls was performed and quantified each time on the same 96-well plate. To quantify target mRNA expression, cDNA copy numbers were calculated based on the results of the standard curve of the same run. Each sample was analyzed in triplicate, and arithmetic means were calculated. The cDNA copy numbers were normalized using the calculated *GAPDH* cDNA copy numbers of the same sample.

1.3. Supplemental Table S2: Primers used in qRT-PCR for absolute quantification

Human Gene		Primer sequence (5′–3′)
SCN5A neonatal	F	CACGCGTTCACTTTCCTTC
	R	ATATTTTCTGATACATACGCCATGAT
SCN5A adult	F	ATCATGGCATACACAACTGAATTT
	R	GAAGGTGCGTAAGGCTGAGA
GAPDH	F	AAGGTGAAGGTCGGAGTCAAC
	R	GGGGTCATTGATGGCAACAATA

2. Results

2.1. Supplementary Figure S1: ECG monitor recordings of the proband

ECG monitor recordings of the proband at the age of 1.5 months showed Torsade de pointes (TdP) and QTcF at 536 ms.

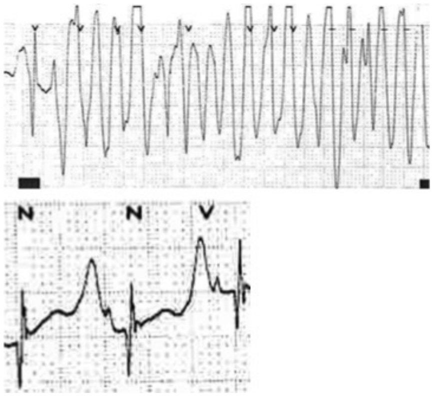

2.2. Supplementary Figure S2: iPSC colonies derived from the lymphoblastoid cell lines (LCLs) of healthy volunteers

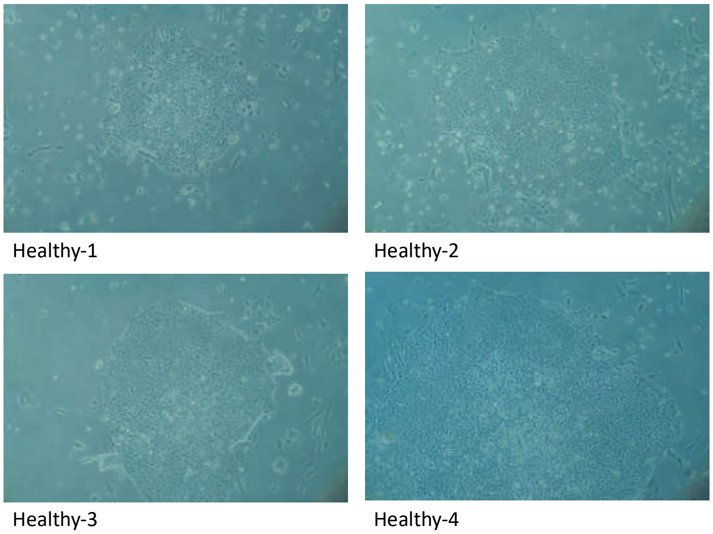

2.3. Supplementary Figure S3: The R1623Q mutation in patient-derived iPSCs was confirmed using DNA sequencing.

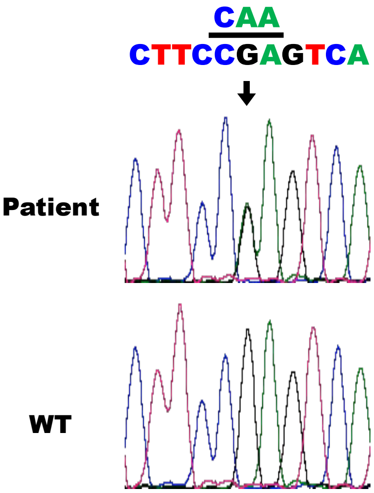

2.4. Supplementary Figure S4: Electrophysiological analysis of induced pluripotent stem cell (iPSC)-cardiomyocytes (CMs) harboring a R1623Q mutation

At concentrations between 1 and 400 nM, isoproterenol, a non-selective beta adrenoreceptor agonist, dose-dependently accelerated the beat rate by up to 159% from the baseline and shortened FPDcF by up to 67% from the baseline in R1623Q mutant-harboring

iPSC-CMs (n = 7, ***p<0.001), indicating that the iPSC-CMs were an intact and coupled beta-adrenergic signalling cascade. Isoproterenol might abbreviate APD (≒FPD) in already prolonged APD consistent with previous study [1].

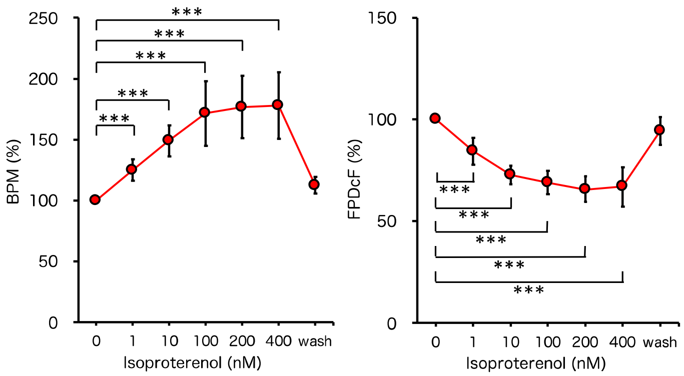

2.5. Supplementary Figure S5: 384 Na current traces of sixth sweep on Data Browser

Sodium currents were measured using a Syncropatch 384 and then analyzed with DataControl 384 PE (Nanion Technologies, München, Germany). This figure shows 384 Na

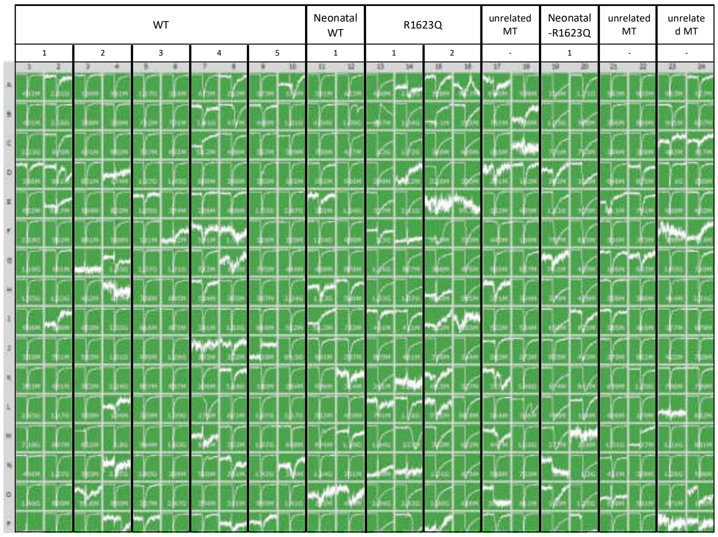

current traces of 6th sweep on Data Browser before quality control. The numbers in the figure are values of their seal resistance. For example, the value "642 M" of column 1A is "642 MΩ".

2.6. Supplementary Figure S6: Expression of the neonatal and adult Nav1.5 mRNA in the adult left ventricle and in the fetal heart

Expression of the neonatal Nav1.5 mRNA compared with those of the "adult" variant was 3.8% in the adult left ventricle (LV, male, 21 yeas of age) and 71.7% in the fetal heart (male, 31 weeks gestation).

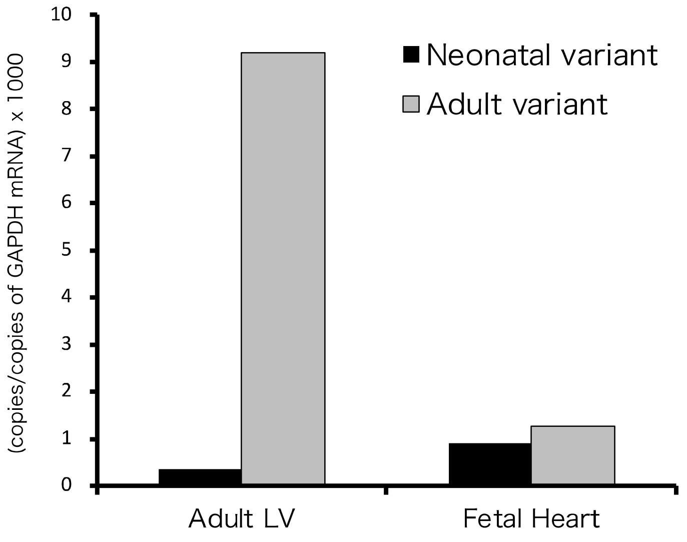

Supplementary references

1. Sutanto, H.; Heijman, J. Beta-Adrenergic Receptor Stimulation Modulates the Cellular Proarrhythmic Effects of Chloroquine and Azithromycin. *Front Physiol* **2020**, *11*, 587709, doi:10.3389/fphys.2020.587709.
